# Supplementary material for: Study of Aroma Characteristics and Establishment of Flavor Molecular Labels in Fermented Milks from Different Fermentation Strains
Source: Foods. 2025 Jun 25;14(13):2237. doi: 10.3390/foods14132237 (PMC12248424; doi:10.3390/foods14132237)
Supplement: Supplementary file 1 [file foods-14-02237-s001.zip › foods-3696855-supplementary.pdf]

1

Table S1. General table of odor compounds.

No.	Compounds	CAS	RI[a]	Odor[b]	N	A2	L1	L2	L3	L4	P1	P2	P3	P4	P5	P6	P7	S1	S2	S3	S4	S5	S7	S8	S9	S10	S11	Identification method[c]
													Alcohols															
1	Ethanol	64-17-5	900	Alcohol	74.335±1.573a	13.219±0.17b	4.886±0.146c	-	-	1.555±0.008i	-	-	2.496±0.005efg	13.429±0.165b	-	-	3.115±0.017de	3.515±0.013d	5.203±0.228c	1.937±0.01ghi	-	2.363±0.008fgh	-	-	-	-	1.773±0.007hi	MS/RI
2	tert-Butanol	75-65-0	920	Camphor	-	-	-	-	-	-	-	-	-	8.591±0.171a	7.981±0.126b	-	-	-	-	-	-	-	-	3.13±0.202c	-	-	-	MS/RI
3	Isopropyl Alcohol	67-63-0	932	Mouldy	0.804±0.002	-	-	-	-	-	-	-	-	-	-	-	-	-	-	-	-	-	-	-	-	-	-	MS/RI
4	Butanol	71-36-3	1154	Whisky	9.685±0.071	-	-	-	-	-	-	-	-	-	-	-	-	-	-	-	-	-	-	-	-	-	-	MS/RI
5	3-Methyl-3-bute	763-32-6	12	fruity	-	5.397±0.	-	-	2.912±0.	-	-	1.749±0.	-	6.553±0.	-	-	-	-	-	-	-	-	1.72±0.0	-	6.983±0.	-	-	MS/RI

	n-1-ol		40			044d			043e			004f		029b									04f		022a			
6	1-Pentanol	71-41-0	1252	Oily	4.504±0.017a	-	-	-	-	-	-	-	-	-	-	1.466±0.006b	-	-	-	-	-	-	-	-	-	-	1.003±0.01c	MS/RI
7	2-Heptanol	543-49-7	1298	lemon	-	-	-	0.576±0.003	-	-	-	-	-	-	-	-	-	-	-	-	-	-	-	-	-	-	-	MS/RI
8	Isopentenol	556-82-1	1301	Fruity	-	-	-	0.519±0.003j	1.725±0.005b	-	-	-	1.039±0.011fg	2.208±0.01a	0.197±0.001k	0.137±0l	1.707±0.007b	-	1.033±0.029g	-	0.781±0.037h	0.708±0.017i	1.344±0.026d	1.502±0.061c	1.263±0.012e	1.042±0.006fg	0.754±0.003h	MS/RI
9	Hexanol	111-27-3	1360	Fruity	10.583±0.05a	4.314±0.03b	2.262±0.039gh	1.69±0.013k	2.286±0.003gh	4.175±0.024c	0.859±0.014n	-	1.582±0.051l	-	2.305±0.102gh	0.749±0.004o	-	-	2.223±0.048hi	2.624±0.014f	1.256±0.008m	1.296±0.034m	2.325±0.013g	1.813±0.007j	3.532±0.009d	1.578±0.018l	1.879±0.011j	MS/RI/O
10	Heptanol	111-70-6	1457	Herbal	4.221±0.06a	-	-	-	-	2.021±0.026b	-	-	1.081±0.009e	-	-	-	-	-	-	1.413±0.02c	-	0.789±0.005f	-	-	-	-	-	MS/RI/O
11	2-Ethylhexanol	104-76-7	1490	Citrus	-	-	-	-	-	-	-	-	-	-	-	-	1.693±0.007a	-	-	-	-	-	-	-	-	-	-	MS/RI

12	2-Nonyl alcohol	628-99-9	1530	Fruity	-	-	-	1.474±0.011	-	-	-	-	-	-	-	-	-	-	-	-	-	-	-	-	-	-	-	MS/RI
13	Octanol	111-87-5	1558	Orange	-	1.026±0.006f	-	-	-	0.977±0.012f	-	-	-	1.186±0.002e	1.256±0.015d	-	-	-	2.622±0.113a	1.587±0.026c	-	-	0.893±0.007g	-	2.096±0.006b	-	-	MS/RI
14	1-Nonanol	143-08-8	1640	Floral	3.499±0.017a	0.84±0.008k	2.942±0.036b	1.13±0.011h	1.023±0.008i	1.12±0.004h	-	-	-	1.208±0.02g	1.757±0.006d	-	0.944±0.005j	-	2.081±0.043c	2.099±0.013c	-	-	0.603±0.002l	-	1.573±0.011e	-	-	MS/RI
15	Furfuryl alcohol	98-00-0	1659	Toast	3.876±0.016a	-	-	-	-	-	-	-	-	-	-	-	-	-	-	-	-	-	-	-	-	-	-	MS/RI/O
16	3-Methylhexan-2-ol	2313-65-7	1441	-	-	-	-	-	-	-	-	-	-	-	2.29±0.054	-	-	-	-	-	-	-	-	-	-	-	-	MS
17	2-Methyl-3-pentanol	565-67-3	1239	-	38.451±0.272a	4.146±0.044e	-	-	8.831±0.034b	-	-	-	-	-	-	-	-	-	-	3.94±0.032f	-	-	7.223±0.071c	-	6.791±0.177d	-	-	MS
														Aldehydes														
1	3-Methy	590-8	9	Pea	0.56	-	-	-	-	-	-	-	-	-	-	-	-	-	-	-	-	-	-	-	-	-	-	MS/

8	lbutanal	6-3	16	ch	9±0.004																							RI
19	Hexanal	66-25-1	1097	grassy	1.6±0.006g	2.579±0.015b	1.65±0.033f	-	-	-	-	0.684±0.021k	-	-	-	-	-	2.063±0.028c	1.971±0.032d	0.86±0.011j	4.625±0.041a	1.132±0.021i	-	-	-	4.606±0.007a	1.492±0.016h	MS/RI/O
20	Nonanal	124-19-6	1390	Floral	11.633±0.007a	-	4.546±0.129e	-	-	-	-	0.32±0.001k	2.829±0.001i	-	3.868±0.023g	-	-	4.697±0.049e	5.058±0.186d	2.426±0.027j	-	3.893±0.028g	-	8.664±0.401b	-	4.151±0.008f	-	MS/RI/O
21	Furfural	1998/1/1	1432	Bakery	2.137±0.009a	-	-	-	-	-	-	-	-	-	-	-	-	-	0.74±0.004b	-	-	0.11±0c	-	-	-	-	-	MS/RI
22	(*E*)-2-Octenal	2548-87-0	1437	Cucumber	-	-	-	-	-	-	-	-	-	-	-	-	-	0.584±0.087a	0.619±0.021a	0.457±0.004b	-	0.326±0.005c	-	-	-	-	-	MS/RI/O
23	Benzaldehyde	100-52-7	1530	Almond	-	-	-	-	2.221±0.002c	-	-	-	-	2.033±0.01d	-	1.329±0.002h	1.705±0.005f	-	2.197±0.077c	-	-	1.504±0.004g	-	2.938±0.009a	1.188±0.011i	-	-	MS/RI/O
24	(*E*)-2-Undecenal	2463-77-6	1755	Orange Pee	-	-	-	1.315±0.003a	-	0.274±0e	-	-	0.536±0.004d	-	-	-	-	-	0.856±0.037b	0.673±0.017c	-	-	-	-	-	-	-	MS/RI

25	3-Methyl-2-butenal	107-86-8	1198	l Sweet	-	-	-	-	-	-	-	-	-	-	-	-	-	-	-	-	-	-	5.066±0.05a	4.112±0.087b	-	-	0.629±0.004c	MS
ketone																												
26	Acetone	67-64-1	808	Fruity	33.238±0.082b	20.025±0.348j	22.322±0.268i	10.703±0.151n	4.407±0.074r	29.187±0.301d	11.383±0.201m	11.347±0.115m	27.056±0.094f	31.19±0.121c	12.971±0.192k	12.323±0.039l	32.686±0.152b	6.22±0.042q	9.825±0.35o	8.094±0.352p	30.874±0.433c	25.923±0.21g	22.692±0.194i	24.284±0.037h	22.305±0.091i	28.375±0.183e	26.562±0.281f	MS/RI/O
27	2-Butanone	78-93-3	904	Fruity	4.435±0.019h	30.417±0.385a	4.533±0.126h	1.511±0.014m	2.215±0.006kl	4.915±0.17g	2.522±0.033jk	2.372±0.011jk	6.287±0.02e	-	-	0.587±0.003n	1.912±0.026l	-	2.472±0.088jk	-	9.122±0.107d	9.377±0.089d	11.822±0.021c	14.676±0.646b	2.666±0.01j	5.542±0.006f	5.487±0.26f	MS/RI
28	2-Pentanone	107-87-9	945	Sweet	20.542±0.083a	-	-	6.959±0.122c	-	-	-	7.108±0.06b	-	-	-	-	-	-	-	-	-	-	-	-	-	-	-	MS/RI
29	2,3-Butanedione	431-03-8	975	Butter	-	65.266±0.309h	49.208±1.263i	-	31.166±0.202lm	11.233±0.086r	103.014±0.224d	-	73.227±0.919g	162.356±1.676b	97.63±0.472e	146.637±0.256c	80.406±0.491f	17.744±0.01p	30.119±0.663m	36.868±0.135k	23.107±0.457o	16.127±0.041q	31.833±0.311	16.798±0.631pq	37.561±0.282k	-	26.086±0.302n	MS/RI/O
30	2-Methyl-3-pentanone	565-69-5	1000	Mint	-	-	-	-	-	-	-	-	-	2.001±0.009	-	-	-	-	-	-	-	-	-	-	-	-	-	MS/RI

31	3-Methyl-2-pentanone	565-61-7	1014	-	-	-	-	-	-	-	-	-	-	1.474±0.038b	2.176±0.019a	-	-	-	-	-	-	-	-	-	-	-	-	MS/RI
32	2,3-Pentanedione	600-14-6	1060	buttery	2.143±0.009l	19.021±0.097c	24.559±0.51b	0.482±0.001m	19.172±0.096c	5.692±0.03h	-	-	14.309±0.202d	-	12.425±0.201f	-	5.605±0.007h	4.86±0.096i	4.02±0.163j	7.467±0.077g	-	3.439±0.011k	7.52±0.072g	5.809±0.465h	12.964±0.069e	-	7.472±0.059g	MS/RI/O
33	3-Heptanone	106-35-4	1130	fruity	-	-	0.934±0.002c	0.929±0.024cd	-	0.972±0.006b	-	1.109±0.013a	-	-	1.092±0.044a	-	0.895±0.032e	-	-	0.792±0.005f	-	-	-	-	0.904±0.005de	0.903±0.01e	-	MS/RI
34	2-Heptanone	110-43-0	1180	Fruity	70.253±0.293a	49.006±0.425e	31.944±0.016op	51.425±0.341d	67.938±0.309b	32.906±0.201no	45.424±1.003gh	45.611±0.613g	36.865±0.285m	44.818±0.678gh	51.364±0.519d	43.993±0.065hi	47.334±0.187f	33.419±0.327n	39.995±1.446jk	31.036±0.29p	26.991±0.262q	28.213±0.12q	47.449±0.807f	43.076±2.143i	41.363±0.409j	37.987±0.085lm	31.613±0.311op	MS/RI/O
35	4-Octanone	589-63-9	1222	-	-	-	1.435±0.016f	1.639±0.016d	-	1.165±0.005j	-	1.3±0.052h	1.419±0.009f	1.862±0.027c	1.37±0.014g	1.206±0.003i	1.601±0.016d	-	-	2.066±0.055b	-	-	-	-	1.498±0.032e	2.169±0.003a	1.047±0.002k	MS/RI
36	3-Hydroxy-2-butanone	513-86-0	1271	Creamy	84.031±0.624k	128.576±1.132f	87.983±1.397j	6.167±0.062q	122.402±0.976g	19.648±0.162p	34.939±0.359o	1.498±0.007r	143.011±1.004c	304.782±2.691a	137.924±1.076d	36.488±0.334o	186.737±1.273b	116.506±0.309h	109.801±2.915i	50.421±0.475n	-	76.03±4.307l	127.144±5.042f	107.973±2.319i	63.423±0.613m	75.278±0.029l	74.115±1.153l	MS/RI/O
37	Hydroxyaceton	116-09-6	12	Caram	1.338±0.	-	-	0.497±0.	-	0.329±0.	-	-	-	-	-	-	0.457±0.	-	-	0.302±0.	-	-	-	-	-	-	-	MS/RI

	e		75	el	005a			003b		004d							004c			004e								
38	Methylheptenone	110-93-0	1341	Citrus	0.711±0.004	-	-	-	-	-	-	-	-	-	-	-	-	-	-	-	-	-	-	-	-	-	-	MS/RI
39	2-Hydroxy-3-pentanone	5704-20-1	1361	Nutty	11.822±0.021a	2.234±0.019o	2.366±0.081n	-	5.231±0.012e	0.828±0.004q	-	-	5.619±0.066d	5.631±0.054d	3.745±0.02g	-	3.716±0.033gh	3.323±0.022i	3.636±0.004h	2.658±0.011	3.089±0.024j	2.485±0.037m	6.434±0.112c	1.669±0.041p	4.725±0.035f	2.943±0.065k	6.443±0.039c	MS/RI
40	2-Nonanone	821-55-6	1385	Sweet	21.944±0.112d	13.384±0.142i	7.566±0.192o	26.024±0.148a	15.362±0.551g	5.551±0.03q	2.84±0.063s	3.908±0.045r	8.659±0.053n	24.646±0.189b	7.658±0.091o	11.947±0.041k	16.823±0.148e	11.266±0.007l	12.385±0.216j	6.438±0.092p	11.109±0.122l	9.314±0.011m	14.018±0.223h	11.75±0.435k	22.804±0.12c	11.739±0.027k	7.338±0.061o	MS/RI/O
41	2-Methyltetrahydrothiophen-3-one	13679-85-1	1510	Fruity	-	-	4.487±0.149fg	-	5.757±0.018d	-	-	-	4.334±0.043g	5.328±0.053e	-	-	7.652±0.079c	4.073±0.014h	8.164±0.286b	3.207±0.039k	-	4.527±0.018f	3.713±0.019i	-	-	-	-	MS/RI
42	Methyl nonyl ketone	112-12-9	1599	Sweet	4.088±0.061a	2.735±0.007f	1.888±0.014k	2.83±0.021e	-	1.344±0.019m	-	-	2.075±0.118i	3.029±0.014d	2.72±0.03f	1.546±0.003l	3.169±0.006c	2.296±0.024h	2.714±0.052f	2.101±0.016i	-	2.001±0.012j	-	-	3.993±0.016b	3.145±0.001c	-	MS/RI/O
43	2(5H)-furanone	497-23-4	176	Butter	1.965±0.004	-	-	-	-	-	-	-	-	-	-	-	-	-	-	-	-	-	-	-	-	-	-	MS/RI

			7																									
44	Ethyl isobutyl ketone	623-56-3	1123	-	-	-	-	-	-	-	-	-	-	-	-	0.683±0.011	-	-	-	-	-	-	-	-	-	-	-	MS
45	3-Methyl-2-heptanone	2371-19-9	1201	Fruity	2.582±0.013l	3.363±0.033h	2.908±0.04j	-	3.874±0.003f	2.962±0.04ij	2.628±0.072kl	2.946±0.018ig	4.302±0.042de	4.273±0.012e	3.048±0.07i	0.994±0.013n	4.756±0.048c	-	4.74±0.187c	3.285±0.009h	4.222±0.04e	4.409±0.017d	-	1.936±0.078m	2.976±0.13ij	3.576±0.028g	2.709±0.018k	MS/O
Esters																												
46	Ethyl acetate	141-78-6	878	Fruit	0.554±0.006	-	-	-	-	-	-	-	-	-	-	-	-	-	-	-	-	-	-	-	-	-	-	MS/RI
47	Butyl acrylate	141-32-2	1175	Fruity	-	-	2.58±0.03	-	-	-	-	-	-	-	-	-	-	-	2.41±0.068b	1.534±0.019c	-	-	-	-	-	-	-	MS/RI/O
48	Butyl Butyrate	109-21-7	1212	Pineapple	-	-	-	-	-	-	-	-	-	-	-	-	-	-	-	0.183±0.001	-	-	-	-	-	-	-	MS/RI
49	γ-octanolactone	698-76-0	1988	Creamy	3.902±0.08a	-	0.58±0.03b	-	-	0.534±0.004c	-	-	0.326±0.003d	-	-	-	-	-	-	-	-	-	-	-	-	-	-	MS/RI
5	γ-decala	705-8	2	Cre	9.70	-	4.72	1.23	1.50	1.63	-	-	1.74	-	-	-	-	1.54	1.27	1.45	1.36	1.68	-	1.84	-	-	-	MS/

0	ctone	6-2	192	amy	9±0.056a		8±0.118b	9±0.009k	7±0.013hi	±0.01fg			±0.016e					4±0.007gh	9±0.048jk	4±0.025i	3±0.009j	7±0.013ef		8±0.136d				RI
51	γ-dodecalactone	2721-22-4	2701	Creamy	-	-	-	-	-	-	-	-	-	-	-	-	-	-	-	-	-	-	1.356±0.012	-	-	-	-	MS/RI
52	Octyl formate	112-32-3	1534	Orange	4.582±0.028a	-	-	-	1.301±0.007b	-	-	-	-	-	-	-	0.963±0.003c	-	-	-	-	-	-	0.788±0.024d	-	-	-	MS
													acids															
53	Acetic acid	64-19-7	1429	sour	108.354±0.944e	68.383±1.078g	-	-	388.631±1.401b	19.212±0.145n	38.776±0.803k	14.999±0.071o	72.56±1.563f	714.208±0.47a	40.316±0.741k	17.169±0.053n	342.032±1.864d	68.959±1.176g	72.315±0.859f	33.052±0.229l	56.363±0.99i	42.94±0.158j	57.935±0.793i	43.131±2.624j	38.506±0.136k	65.388±0.192h	26.346±0.272m	MS/RI/O
54	Propionic acid	1979/9/4	1508	cheese	-	-	-	-	-	-	-	-	-	-	-	-	-	-	-	-	-	-	-	-	-	1.005±0.004	-	MS/RI
55	Isobutyric acid	79-31-2	1581	Sour	2.957±0.017a	-	-	-	-	-	-	-	-	0.653±0.003b	-	-	-	-	-	-	-	-	-	-	-	-	-	MS/RI
56	Butanoic acid	107-92-6	16	Chees	15.763±0	17.689±0	46.918±0	22.503±	57.754±0	10.486±0	2.964±0.	1.216±0.	61.448±0	40.307±0	14.177±0	1.85±0.0	49.102±0	37.086±1	38.169±0	19.322±	-	60.416±0	39.463±0	35.21±0.	24.094±	72.008±1	45.404±0	MS/RI/

			30	e	.005q	.099p	.516g	0.233n	.106e	.218s	035t	009u	.358c	.385i	.116r	07u	.488f	.043k	.421j	0.329o		.386d	.614i	366l	0.205m	.386a	.49h	O
57	Isovaleric acid	503-74-2	1680	Sweaty	33.462±0.118a	-	-	3.03±0.032b	-	-	-	-	-	-	1.911±0.021c	-	-	-	-	-	-	-	-	-	-	-	-	MS/RI
58	Valeric acid	109-52-4	1756	Sweaty	1.038±0.001g	-	1.669±0.017b	0.529±0.005k	-	0.768±0.005i	-	-	1.435±0.003d	0.665±0.009j	-	-	0.983±0.006h	1.32±0.002f	1.314±0.034f	0.647±0.007j	-	1.45±0.04cd	0.487±0.015l	-	-	3.083±0.002a	1.346±0.019e	MS/RI
59	Hexanoic acid	142-62-1	1810	Sweaty	102.625±0.404j	54.557±0.237n	141.658±1.391e	85.315±1.293l	127.545±0.522g	-	17.709±0.395p	13.494±0.034q	164.153±1.538c	111.615±2.772h	45.224±0.55o	15.744±0.085pq	140.003±0.728e	-	130.854±4.492g	81.221±0.882m	135.064±0.205f	179.962±2.193b	96.285±1.381k	102.944±2.391j	100.791±0.311j	201.721±2.221a	106.983±1.059i	MS/RI/O
60	Heptanoic acid	111-14-8	1950	sweaty	3.469±0.016h	1.298±0.001q	4.363±0.035b	2.545±0.022k	1.678±0.008p	2.441±0.015l	0.658±0.011r	0.469±0.001s	3.931±0.006d	2.416±0.034l	-	0.224±0.002t	3.74±0.011f	3.819±0.044e	3.528±0.039g	2.595±0.013k	3.802±0.015e	4.641±0.013a	1.744±0.027o	2.051±0.034m	1.885±0.036n	-	3.18±0.038i	MS/RI
61	Octanoic acid	124-07-2	2038	cheese	83.934±0.415a	34.288±0.261k	59.196±1.001d	50.14±0.08f	44.269±0.299h	38.839±0.159i	13.443±0.264m	11.591±0.339n	58.719±0.561d	46.674±1.007g	26.352±0.287l	10.289±0.373n	59.771±0.05d	-	49.999±1.822f	39.095±0.027i	-	75.671±2.66b	36.847±0.329j	44.702±1.07h	38.434±0.123ij	84.365±0.179a	45.905±0.041gh	MS/RI
62	Nonanoic acid	112-05-0	2144	Cheese	12.802±0.073a	9.894±0.114c	4.983±0.011l	8.606±0.181d	5.175±0.021k	7.458±0.057g	-	4.019±0.028n	5.357±0.029ij	5.209±0.084jk	4.569±0.066m	2.404±0.004o	3.973±0.095n	-	-	4.658±0.056m	7.732±0.133f	6.018±0.041h	4.834±0.048l	5.375±0.143i	11.28±0.077b	5.322±0.01ijk	-	MS/RI

63	Decanoic acid	334-48-5	2279	Unpleasant	33.949±0.108a	7.126±0.033m	17.849±0.281e	10.826±0.071i	7.38±0.054m	17.868±0.124e	-	5.06±0.023p	12.197±0.058g	9.475±0.072k	6.391±0.087n	2.758±0.017q	14.886±0.063f	11.594±0.051h	11.767±0.436h	9.991±0.127j	-	19.769±0.035c	5.655±0.125o	8.466±0.384l	4.98±0.034p	24.429±0.018b	18.286±0.032d	MS/RI
64	Benzoic acid	65-85-0	2436	Ester aroma, ammonia aroma	-	-	-	-	-	-	2.832±0.078	-	-	-	-	-	-	-	-	-	-	-	-	-	-	-	-	MS/RI
65	2-Methylhexanoic acid	4536-23-6	1698	mint	-	-	-	-	-	-	-	-	-	1.272±0.007	-	-	-	-	-	-	-	-	-	-	-	-	-	MS
Sulfur-containing compounds																												
66	Dimethyl disulfide	624-92-0	1080	Onion	-	1.113±0.007b	-	-	1.317±0.021a	-	-	-	-	-	-	-	-	-	-	-	-	-	-	-	-	-	-	MS/RI/O
67	Dimethyl trisulfid	3658-80-8	1137	sulfur	-	-	-	-	1.449±0.001a	-	-	-	-	-	-	0.227±0.003b	-	-	-	-	-	-	-	-	-	-	-	MS/RI/O

	e		8																									
68	Dimethyl sulfone	67-71-0	1895	sulfur	12.111±0.069b	9.15±0.098d	5.218±0.106j	8.877±0.049e	3.789±0.026m	2.669±0.02o	6.4±0.103h	4.333±0.119kl	2.408±0.038p	5.908±0.12i	8.084±0.1f	4.399±0.013k	5.989±0.035i	6.458±0.14h	4.165±0.146l	3.125±0.074n	3.08±0.005n	1.626±0.006r	3.069±0.113n	5.435±0.217j	16.794±0.139a	6.858±0.293g	2.156±0.041q	MS/RI
69	Dimethyl sulfide	75-18-3	<800	cabbage	-	-	0.858±0.014c	-	-	1.195±0.012b	-	-	3.657±0.069a	-	-	-	-	-	-	-	-	-	-	-	-	1.218±0.003b	1.217±0.001b	MS/O
												Other compounds																
70	2-Pentylfuran	3777-69-3	1229	soymilk	2.8±0.009a	-	-	-	-	-	-	-	-	-	-	-	-	-	-	-	-	-	-	-	-	-	-	MS/RI/O
71	Styrene	100-42-5	1254	Plastic	-	-	-	-	-	0.933±0.001c	-	-	-	-	-	-	3.111±0.006b	-	-	-	-	-	-	4.431±0.08a	-	-	-	MS/RI
72	Phenol	108-95-2	1985	Plastic	2.13±0.019a	-	0.302±0.008f	0.399±0.003b	-	0.255±0.001g	-	0.257±0g	-	-	-	-	-	-	0.317±0.004e	0.37±0.02c	-	0.337±0.003d	-	-	-	-	-	MS/RI
73	4-Methylphenol	106-44-5	2060	Green-like	3.537±0.017	-	-	-	-	-	-	-	-	-	-	-	-	-	-	-	-	-	-	-	-	-	-	MS/RI

74	(+)-Limonene	5989-27-5	1195	Citrus	-	-	-	-	-	-	-	-	-	1.496±0.051b	-	-	-	-	-	-	-	-	-	-	-	-	-	MS

a Retention indices. b Odor quality as perceived at the sniffing port. c MS, RIand O were represented for identifying by mass spectra (MS), retention indices (RI), olfactometry (O), respectively. d Different letters in the same rows indicate significant differences ($P \leq 0.05$). “-”means that this compound was not detected in this sample.
